# Ultrasonic surgical and electrosurgical system (USES) with conventional ultrasonic scalpel (CUS) in gastrectomy: a retrospective cohort study

**DOI:** 10.1186/s12962-022-00344-5

**Published:** 2022-05-07

**Authors:** Changqing Jing, Yuezhi Chen, Liang Shang, Jinshen Wang, Guodong Lian, Feng Tian, Yixue Shao, Yingnan Zhao, Jianwei Xuan, Leping Li

**Affiliations:** 1 Department of Gastrointestinal Surgery, Shandong Provincial Hospital Affiliated to Shandong First Medical University, Jinan, 250021 China; 2 Department of Health Policy and Management, School of Public Health and Tropical Medicine, Tulane University, New Orleans, LA USA; 3 Division of Clinical and Administrative Sciences, College of Pharmacy, Xavier University of Louisiana, New Orleans, LA USA; 4 Sun Yat-Sen University, Guangzhou, China

**Keywords:** Harmonic ACE +7, Cost analysis, Gastrectomy, Gastric cancer

## Abstract

**Background:**

Harmonic ACE +7 Shears with Advanced Hemostasis is an upgraded ultrasonic device, an ultrasonic surgical and electrosurgical system (USES). The study aimed to evaluate the economic and clinical effectiveness of the USES compared with the conventional ultrasonic scalpel (CUS) in gastrectomy.

**Methods:**

We conducted a single-center, retrospective cohort study using the electronic medical records in China. We collected intraoperative and postoperative data from gastric cancer patients who underwent the endoscope-assisted distal gastrectomy from 2018 to June 30, 2019. Procedure-related costs were estimated. We used linear regression by controlling a set of covariates to assess the effect of USES on outcomes.

**Result:**

Out of 87 eligible patients, the USES group (40 patients) and CUS group (47 patients) were comparable in terms of age, medical history and stages of cancer. Compared with the CUS, the USES saved 4.27 hemoclips per person (95% CI 0.57–7.97, p < 0.05) and 34.18 ml intraoperative blood per person (95% CI 8.74–59.62 ml, p < 0.05), respectively. Postoperative length of stay (LOS) was shorter in the USES group (7.90 ± 1.95 vs. 9.26 ± 2.81 days) but the difference was not statistically significant (p = 0.05).

**Conclusions:**

The USES group was associated with fewer hemoclips use and intraoperative blood loss in patients undergoing laparoscopic gastrectomy at comparable costs.

**Supplementary Information:**

The online version contains supplementary material available at 10.1186/s12962-022-00344-5.

## Background

Gastric cancer (GC) is the third leading cause of cancer deaths worldwide, with an estimated 783,000 deaths, and has the fifth-highest incidence among cancers [1]. Over a million new cases of gastric cancer each year are diagnosed worldwide [2]. More than 70% of the new gastric cancer cases occur in developing countries [3]. China is one of the high-burden areas in the world, with an estimated 456,124 new cases in 2018 [4]. Current treatment guidelines recommend the use of surgical gastric resection for the management of resectable GC [5]. Compared with open surgery, laparoscopic gastrectomy for early-stage gastric cancer has been widely accepted in the world because of its advantages in terms of reduced intraoperative blood loss, reduced postoperative pain, and accelerated recovery without compromising the survival [6–8]. The gastrectomy requires exhaustive hemostasis with a dry operative field for high-quality lymph node dissection and to avoid inadvertent damage to important structures such as the pancreas [9]. In recent years, new hemostatic tools have been developed with the advent of laparoscopic surgery. Ultrasonic scalpel has been used in several surgeries, such as hysterectomy, colectomy, thyroid, and gastrectomy surgery [9–12]. According to a previous meta-analysis, the ultrasonic scalpel has higher performance in cutting, hemostatic, and decreasing the intraoperative blood loss and operative time, compared with the conventional technique for hemostasis during gastrectomy including ligation and electrosurgery [13, 14]. In addition, better performances were found in the ultrasonic scalpel group over the conventional group regarding postoperative hospitalization days, abdominal drainage volume, and time for recovery of gastrointestinal functions.

Conventional ultrasonic scalpel is only capable of sealing vessels up to 5 mm in diameter. Vessels with 5–7 mm diameter are usually sealed by advanced bipolar vessel sealing technologies, hemostatic clips, or suture ligation. Harmonic ACE +7 Shears with Advanced Hemostasis (Harmonic ACE +7)is an upgraded ultrasonic device that leverages adaptive tissue technology with the addition of predictive analytics to modulate energy delivery during the sealing cycle, which can seal vessels up to 7 mm in diameters with burst pressures significantly greater than those observed with advanced bipolar technologies [15]. As the first purely ultrasonic device with a 7 mm sealing indication, the efficacy of this new device has been evaluated in several human clinical trials [16–18]. The use of this device could potentially improve operative efficiency by eliminating the need for instrument exchanges during surgery, and this device is best suited for surgeries that require dissection, mobilization, and large vessel sealing. While this device has potential intraoperative and postoperative benefits over conventional devices, the costs related to surgery and hospitalization need to be rigorously evaluated. No economic evaluation studies have been published for Harmonic ACE +7 using real-world data.

The USES was the first disposable ultrasonic scalpel in China during our study period and its related operation cost was much higher than the cost of traditional device. At that time, only Shandong had reimbursement policy to support the use of this new surgical technology. According to 2016 Disease and Health Report in Shandong, the gastric cancer was the second most common cancer in the province. Shandong Provincial Hospital recorded items that we need for this study and these items were not always captured in other hospitals. Therefore, this study aimed to investigate operative and economic outcomes of patients who underwent laparoscopic-assisted distal gastrectomy with ultrasonic surgical and electrosurgical system (Harmonic ACE +7, USES group) versus conventional ultrasonic scalpel (CUS group) in a real-world institution in Shandong, China.

## Methods

This retrospective cohort study used the electronic medical records from the Gastrointestinal Surgery Department at Shandong Provincial Hospital in China. Our population consisted of gastric cancer patients (>=18 years old) who underwent the laparoscopic-assisted distal gastrectomy from January 1, 2018 to June 30, 2019. No other exclusion criteria were set. The study followed the Strengthening the Reporting of Observational Studies in Epidemiology (STROBE) guidelines [19].

Classification of these patients was based on two types of ultrasonic energy techniques. The choice of ultrasonic scalpel was made based on the surgeon’s preference. The surgeons are from the same gastrointestinal surgery group. All of them have over 8 years of clinical experience and used consistent standards in clinical diagnoses and treatment. The ultrasonic surgical and electrosurgical system (USES) group was patients who received the surgery using Harmonic ACE +7, and the conventional ultrasonic scalpel (CUS) group was those who had their procedures using a conventional ultrasonic scalpel. For both groups, the demographic characteristics and diagnostic information were extracted from the electronic medical records including age, gender, height, weight, body mass index (BMI), smoking status, medical conditions, and cancer stage.

Intraoperative clinical outcomes included the number of hemoclips used during the surgery, intraoperative blood loss, and operative time. Postoperative clinical outcomes include postoperative length of stay (LOS) and severe postoperative complications. Severe postoperative complications were defined as postoperative abdominal hemorrhage or re-operation. The cost difference between USES and CUS groups was assessed by comparing the ultrasonic technique utilization-related costs.

We used resource-based costing to assess essential costs of USES versus CUS based on a clinical pathway recommended by expert consensus [20]. Resource utilization in the following sections was considered: intraoperative and postoperative utilization in hospitals. We excluded the preoperative costs because there was no significant difference between the two groups in preoperative care according to recommendations from three clinical experts. For intraoperative costs, we considered the costs related to hemostatic devices and supplies. We focused on the costs of ultrasonic scalpel and costs of hemoclips utilization during surgery. We assumed other intraoperative items were the same between the two groups, such as anesthesia and other surgical consumables (e.g., surgical sutures, staplers), which were unrelated to alternative ultrasonic techniques. For postoperative costs, we considered the postoperative LOS and the daily essential costs of postoperative hospitalization care. The list of daily essential items of postoperative hospitalization care and the number of daily items used were provided and verified by three clinical surgeons based on general clinical practice. The costs of daily essential items during postoperative hospitalization included treatment fee, bed fee, drug fee, and diagnosis and examination fee (Additional file 1: Table S1). The unit price of ultrasonic scalpels, hemoclips, and items of daily postoperative care was extracted from the hospital’s fee schedule for 2019.

Descriptive analyses were reported as mean (standard deviation) for continuous variables, or count and percentage for categorical variables. We applied the Kruskal–Wallis test or t-test when appropriate for continuous variables and the Chi-Square test for categorical variables to determine whether the differences observed across the two groups were significant. In situations where the sample size was insufficient to perform a Chi-Square test (less than 5 observations per cell), the Fisher Exact test was performed. To assess the effect of USES on outcomes, we performed the linear regressions,controlling for age, gender, height, weight, BMI, smoking status, medical conditions, and cancer stage. The p values less than 0.05 were considered statistical significance (two-tailed test).

This study was approved by the Institutional Review Board of Shandong Provincial Hospital. All procedures performed in this study involving human participants were following the ethical standards of the institutional and/or national research committee and with the 1964 Helsinki Declaration and its later amendments or comparable ethical standards. Informed consent was obtained from all individual participants included in the study.

## Results

We included 87 patients, out of whom 47 patients received the CUS and 40 received the USES (Table 1). The two groups were comparable on demographic and clinical characteristics, including age, gender, height, weight, BMI, smoking status, medical conditions, and cancer stage (all p values > 0.05).

**Table 1:** Baseline characteristics of patients undergoing the laparoscopic assisted distal gastrectomy with CUS or USES

	CUS	USES	p-value
Sample size	47	40	–
Age (years)	59.30 ± 11.18	58.30 ± 9.15	0.654
Gender, N (%)			1.000
Male	33 (71.70%)	28 (70.00%)	
Female	13 (28.30%)	12 (30.00%)	
Height (cm)	166.89 ± 7.02	169.75 ± 6.20	0.059
Weight (kg)	65.00 ± 9.54	67.36 ± 9.01	0.260
BMI (kg/m^2^)	23.29 ± 2.74	23.39 ± 3.02	0.873
Smoking, N (%)	18 (39.10%)	15 (38.50%)	1.000
Medical condition, N (%)			
Hypertension	15 (31.90%)	7 (17.90%)	0.219
Diabetes	6 (13.00%)	3 (7.70%)	0.656
Heart diseases	3 (6.50%)	5 (13.20%)	0.511
Arrhythmia	3 (6.80%)	1 (2.60%)	0.697
Cancer stage, N (%)			0.528
Stage I	16 (42.10%)	18 (51.40%)	
Stage II	8 (21.10%)	3 (8.60%)	
Stage III	11 (28.90%)	11 (31.40%)	
Stage IV	3 (7.90%)	3 (8.60%)	

Percentages were calculated based on non-missing values

*BMI* body mass index, *CUS* conventional ultrasonic scalpel, *USES* ultrasonic surgical and electrosurgical system

We compared the intraoperative and postoperative clinical outcomes between the groups (Table 2). The USES group was significantly associated with a decrease of 4.27 (95% CI 0.57–7.97, p < 0.05) in hemoclips use, compared with the CUS group. The USES decreased the mean intraoperative blood loss by 34.18 ml (95% CI 8.74–59.62 ml, p < 0.05). Although the mean operative time favored the USES group over in the CUS group (184.47 ± 60.12 vs. 205.00 ± 62.59 min), the adjusted difference was not statistically significant (p = 0.10). No significant decrease was found in mean postoperative LOS. No severe postoperative complications were reported in either the CUS group or the USES group.

**Table 2:** The clinical outcomes of the USES group versus CUS group

	CUS	USES	Adjusted difference: USES vs CUS	
	Mean (SD)	Mean (SD)	Estimates (95% CI)	p-value
Number of hemoclips	23.40 (7.80)	19.00 (5.90)	− 4.27 (− 7.97, − 0.57)	< 0.05
Intraoperative blood loss (ml)	103.56 (57.77)	61.62 (30.98)	− 34.18 (− 59.62, − 8.74)	< 0.05
Operative time (min)	205.00 (62.59)	184.47 (60.12)	− 27.55 (− 59.34, 4.24)	0.10
Postoperative length of stay (days)	9.26 (2.81)	7.90 (1.95)	− 0.89 (− 0.79, 1.00)	0.05
Postoperative severe complications (n [%])				
Yes	0 [0]	0 [0]	–	–
No	47 [100%]	40 [100%]		

Adjusted difference: the coefficient of treatment indicator (USES vs. CUS, coded as 1 vs. 0) in a linear regression by controlling age, gender, height, weight, BMI, smoking status, medical conditions, and cancer stage

*CUS* conventional ultrasonic scalpel, *USES* ultrasonic surgical and electrosurgical system, *SD* standard deviation.

The results of cost analysis (Table 3) were based on the 2019 fee scale in hospital; the unit price of a conventional ultrasonic scalpel and the Harmonic ACE +7 was ¥2976 and ¥5004, respectively; the unit price of the hemoclip was ¥89.10; the daily essential cost of postoperative hospitalization was ¥1038.86 **(**Additional file 1: Table S1). The mean cost of hemoclips in the USES group was lower than that in the CUS group (¥1688.44 ± ¥524.98 vs. ¥2083.42 ± ¥ 696.28). Controlling for the covariates, the USES was associated with a decrease in hemoclip costs of ¥380.46 (95% CI ¥50.79–¥710.13, p < 0.05). The mean cost of postoperative hospitalization was lower in the USES group than CUS group (¥8206.99 ± ¥2021.01 vs. ¥9614.98 ± ¥2918.05) but the adjusted difference between the two groups was not statistically significant (p = 0.36). No significant difference was found in total costs between the two groups (adjusted difference: ¥625.30, 95% CI ¥− 695.93–¥1946.53, p = 0.36).

**Table 3:** Ultrasonic technique utilization-related cost of USES group versus CUS group

	CUS	USES	Adjusted difference: USES vs CUS	
	Mean (SD)	Mean (SD)	Estimates (95% CI)	p-value
Intraoperative costs (¥)				
Ultrasonic scalpel^a^	2976.00	5004.00	–	–
Hemoclips^b^	2083.42 (696.28)	1688.44 (524.98)	− 380.457 (− 710.127, − 50.787)	< 0.05
Postoperative costs (¥)				
Postoperative hospitalization^c^	9614.98 (2918.05)	8206.99 (2021.01)	− 924.59 (− 820.70, 1038.86)	0.05
Total costs^d^ (¥)	14,674.40 (3095.97)	14,899.44 (2044.20)	625.30 (− 695.93, 1946.53)	0.36

Adjusted difference: the coefficient of treatment indicator (USES vs. CUS, coded as 1 vs. 0) in a linear regression by controlling age, gender, height, weight, BMI, smoking status, medical conditions, and cancer stage

*CUS* conventional ultrasonic scalpel, *USES* ultrasonic surgical and electrosurgical system, *SD* standard deviation

^a^The unit price was ¥2976.00 for a conventional ultrasonic scalpel and ¥5004.00 for Harmonic ACE +7. All patients used one ultrasonic scalpel during surgery.

^b^The unit price of hemoclips was ¥89.10. The cost of hemoclips per patient was calculated by the number of hemoclips used × unit price of hemoclips.

^c^The daily essential cost of postoperative hospitalization was ¥1038.86 (Additional file 1: Table S1). The cost of postoperative hospitalization per patient was calculated by postoperative LOS × daily essential cost of postoperative hospitalization.

^d^Total cost related to ultrasonic technique utilization

## Discussion

We found overall favorable effectiveness of a new advanced ultrasonic device, Harmonic ACE +7 Shears with Advanced Hemostasis for laparoscopic gastrectomy recipient in a real-world practice setting. With the new device, the number of hemoclips and intraoperative blood loss were significantly decreased and might offset the higher technology cost associated with the USES approach. Yet not statistically significant, we also identified shorter operative time and postoperative length of stay in the USES group with no severe postoperative complications found.

To the best of our knowledge, no study has evaluated the clinical outcomes and economic value of this new device, Harmonic ACE +7, compared with the conventional ultrasonic scalpel for gastrectomy recipients in a real-world setting in China. Previous studies compared two quite different approaches, conventional ultrasonic scalpel versus electrocautery or electrosurgery, among open or laparoscopic surgeries [9, 12, 14, 21, 22]. Ultrasonic scalpel has better performance not only on intraoperative outcomes but also on postoperative outcomes. The current study compared two similar technologies, the advanced ultrasonic scalpel (Harmonic ACE +7) and the conventional ultrasonic scalpel, in laparoscopic-assisted distal gastrectomy. Our findings are consistent with several clinical trials for the USES (Harmonic ACE +7) that were performed to evaluate the clinical efficacy of this new device, focusing on hysterectomy, lobectomy, and colectomy [16, 18]. The clinical benefits of Harmonic ACE +7 on preventing intraoperative bleeding have been demonstrated in these surgeries. In forty patients undergoing the laparoscopic colectomy procedure, the first-pass hemostasis of the inferior mesenteric artery was achieved and maintained with no required additional hemostatic measures in all patients and only one adverse event (postoperative anemia) was considered possibly related to the study device [17]. Pulmonary artery branch sealing with an ACE +7 during video-assisted thoracoscopic surgery lobectomy was effective for vessels 7 mm or less and there was no difference in bleeding between ultrasonic-sealed vessels and vessels sealed with endostaplers [16]. For its performance in laparoscopic hysterectomy, 5 of forty patients received additional hemostasis using conventional bipolar or monopolar energy [18]. Without control groups in these trials, we could not know whether Harmonic ACE +7 has better performance than conventional ultrasonic devices in the surgery. The current study has demonstrated the better performance of this new ultrasonic device. As expected, the new device achieved the potential to reduce the need for additional hemostatic devices or products in our study, compared with the conventional ultrasonic scalpel.

Notably, our study is the first study to compare the clinical outcomes and economic value of two ultrasonic technologies in laparoscopic-assisted distal gastrectomy. Although the surgery cost of adopting the Harmonic ACE +7 was higher than the cost associated with the CUS group, the meaningful intraoperative and postoperative benefits for patients receiving the USES had cost savings due to shorter LOS after surgery and nearly offset the higher surgery charge. With parity in total costs between the USES and the CUS, patients would benefit from better clinical outcomes and experience during surgery and postoperative hospitalization.

Furthermore, the significant improvement on hemostasis of the USES would improve operative efficiency by eliminating the need for instrument exchanges during the operation. In the perspective of hospital administrators, by adopting the USES, reductions in operation time and LOS could improve operating room turnover and hospital bed turnover rates. Our findings also have implications that using new ultrasonic techniques might not increase operational and budget pressure of hospitals under disease-related group (DRG) payment.

We highlight several limitations of this study. First, our findings may not have extensive generalizability due to the sample size and being a single-center study. Larger multi-center studies are needed for more evidence. Second, patients in the USES group may be subject to provider-induced demand for treatment items with higher prices. Despite comparable baseline characteristics between the two groups, selection bias might not be eliminated due to unobservable factors. Third, we standardized the daily essential costs during hospitalization, which were verified by three clinical experts. Similar results might be achieved in hospitals where the same type of operations is performed on a similar scale. However, the economic parameters that we used for the present analysis may differ from other sites based on the type of health care system and reimbursement strategies. We expect that the total costs could have larger variations when more heterogeneous patient populations and hospitals are included in future studies. At last, our study only considered the surgery and inpatient costs. We did not have information on costs related to care after being discharged from the hospital.

## Conclusions

Compared with the CUS, the USES usage was associated with better clinical performance and similar costs for laparoscopic gastrectomy recipients. Our findings provide real-world evidence for patients, doctors, and policymakers in decision-making on surgical devices. Further studies are called for with a larger sample size and longer follow-up after gastrectomy.

## Supplementary Information

**Additional file 1: Table S1.** The daily essential costs of postoperative hospitalization care for laparoscopic assisted distal gastrectomy recipient.

## Data Availability

The datasets generated and/or analyzed during the current study are not publicly available but are available from the corresponding author on reasonable request.
