# Supplementary material for: Ultrasonic surgical and electrosurgical system (USES) with conventional ultrasonic scalpel (CUS) in gastrectomy: a retrospective cohort study
Source: Cost Eff Resour Alloc. 2022 May 7;20:21. doi: 10.1186/s12962-022-00344-5 (PMC9078022; doi:10.1186/s12962-022-00344-5)
Supplement: Supplementary file 1 — Additional file 1: Table S1. The daily essential costs of postoperative hospitalization care for laparoscopic assisted distal gastrectomy recipient. [file 12962_2022_344_MOESM1_ESM.docx]

# Supplementary Materials

Table S1. The daily essential costs of postoperative hospitalization care for laparoscopic assisted distal gastrectomy recipient.

Type of cost Medical orders Unit price Number Cost

|  | | (CNY/unit) |  | (CNY) |
| --- | --- | --- | --- | --- |
| Precise filter infuser JMQ | | 11.56/set | 1 | 11.56 |
| Vacuum blood collection tube | | 1.19/unit | 3 | 4.17 |
| Massive dressing change | | 98.00/time | 1/3^a^ | 32.67 |
| Class II nursing care | | 30.00/day | 1 | 30.00 |
| Arterial and venous catheter care | | 6.00/time | 4 | 24.00 |
| Intravenous hyperalimentation therapy (devices included) | | 20.00/time | 1 | 20.00 |
| Treatment | Intravenous drip (bottles) | 1.00/bottle | 5.5 | 5.50 |
|  | Intravenous drip (drug injection) | 4.50/set | 1 | 4.50 |
|  | Intravenous injection | 4.50/time | 2 | 9.00 |
|  | Drainage | 10.00/day | 1.5 | 15.00 |
|  | Pain assessment | 10.00/time | 4 | 40.00 |
|  | Venipuncture blood collection | 4.50/time | 1 | 4.50 |
|  | Disposable sterile syringe | 0.38/time | 3 | 1.14 |
|  | Disposable blood collection needle | 0.32/unit | 1 | 0.32 |
| Bed | Bed | 80.00/day | 1 | 80.00 |
|  | Vitalipid | 11.16/dose | 1 | 11.16 |
|  | Soluvit N | 8.80/dose | 1 | 8.80 |
|  | 0.9% Sodium chloride injection (10ml: 0.09g) | 1.12/bottle | 2 | 2.24 |
|  | Kabiven | 286.38/bag | 1 | 286.38 |
|  | Alanyl Glutamine Injection (Litai) | 152.90/bottle | 1 | 152.90 |
| Drugs Fish oil triglycerides injectable emulsion  (Omegaven） | | 246.35/bottle | 1 | 246.35 |
| 0.9% Sodium chloride injection（100ml: 0.9g) | | 5.73/bag | 1 | 5.73 |
| Concentrated sodium chloride injection | | 2.00/dose | 4 | 8.00 |
| Furosemide injection | | 2.98/dose | 1 | 2.98 |
| Sodium lactate Ringer injection | | 3.97/bag | 1 | 3.97 |
| Diagnosis and Hospitalization diagnosis and examination fee | | 28.00/day | 1 | 28.00 |

examination

**Total (CNY**¥**） 1,038.86**

Notes: a. massive dressing usually changes every 3 days according to the clinical practice.
